# Editorial: Bridging Techniques: Basic Science of Molecules, Cellular Systems, and Whole-Organ Physiology

**DOI:** 10.3389/fphys.2022.879396

**Published:** 2022-03-24

**Authors:** Robert Szulcek, Christopher N. Johnson, James Todd Pearson, Vasco Sequeira

**Affiliations:** ^1^Laboratory of in Vitro Modeling Systems of Pulmonary and Thrombotic Diseases, Institute of Physiology, Charité – Universitätsmedizin Berlin, Berlin, Germany; ^2^Freie Universität Berlin and Humboldt-Universität zu Berlin, Berlin, Germany; ^3^German Heart Center Berlin, Berlin, Germany; ^4^Department of Chemistry, Mississippi State University, Starkville, MS, United States; ^5^Department of Cardiac Physiology, National Cerebral and Cardiovascular Center Research Institute, Osaka, Japan; ^6^Monash Biomedicine Discovery Institute and Department of Physiology, Monash University, Clayton, VIC, Australia; ^7^Comprehensive Heart Failure Center (CHFC), University Clinic Würzburg, Würzburg, Germany

**Keywords:** whole-organ physiology, cardiovascular – methods, cardiac muscle mechanics, *in vitro* cellular systems, non-sarcomeric proteins, sarcomeric apparatus

Understanding cardiovascular physiology and improving cardiovascular disease prevention and/or treatment are tightly intertwined with the rigor of experiments and interdisciplinary interpretation of the data. Experimental rigor is the prerequisite for robust and reproducible data that provide confidence and certainty. Interdisciplinary data interpretation allows for novel perspectives that build upon the data and conclusions of others. Hence, advancing and innovating cardiovascular (patho)physiology requires the continued integration of multiple disciplines, scientific backgrounds, and a detailed understanding of available methodology.

Current routinely implemented methods for quantifying cardiovascular performance involve several layers of complexity in their scope ranging from the whole organism to *ex vivo* organs, individual protein-protein interactions, and genome regulation. Despite or because of the worldwide availability and usage of these fundamental techniques, there exists a vital need to continuously re-evaluate and comprehensively discuss their strengths and limitations. This is especially important when translating/scaling the findings to whole organs or even the entire organism. In this current Research Topic of *Frontiers in Physiology* we were seeking to highlight the advantages and limitations of some of the most frequently applied techniques in cardiovascular and specifically cardiac research.

Proper heart function depends on adaptive changes in load and muscle length during the cardiac contraction-relaxation cycle. Chronic perturbation of proper heart pump function is clinically classified as heart failure. Modulation of the cardiac cycle involves many contributions, including alterations in cellular ion dynamics (*i.e*., excitation-contraction coupling) and changes to the viscoelastic properties of the myocardial wall, such as alterations to sarcomeric (*i.e*., cross-bridges and titin) and non-sarcomeric proteins (*i.e*., microtubules and the extracellular matrix) (Sequeira and van der Velden, 2015). The influence of these alterations on cardiac contraction-relaxation over the cardiac cycle varies in magnitude, duration and timing within the cycle, and thus a comprehensive understanding of these routinely available methodologies to assess their roles in cardiac function is paramount to the understanding of cardiovascular health and disease states (Figure 1).

**Figure 1:**
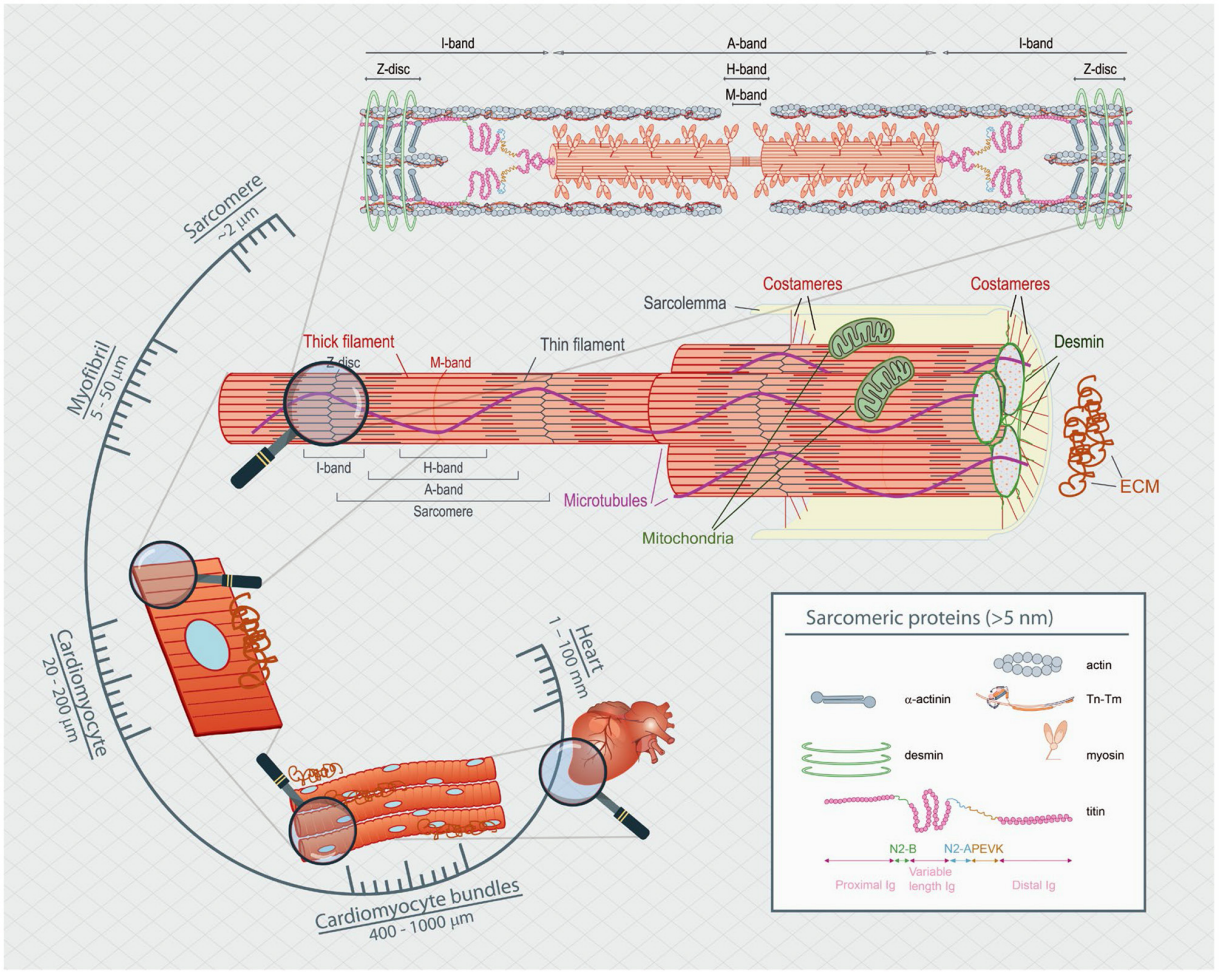
Spatial scaling of the heart's functional components. The heart is composed of many layers of complexity, which span many orders of magnitude. From the nanometer length range of actin-myosin interactions to the micrometer level of the sarcomeric apparatus and up to the hundreds of micrometer of single cells, passing through multicellular sheets of bundles of cells measured in the millimeter length that make up the millimeter rodent animal heart.

In this Research Topic, Knight et al. elaborate on the strengths and weaknesses of five of the most commonly used *in vitro and ex-vivo* techniques to understand cardiac muscle mechanics. The simplicity offered by solution systems with purified actin and myosin provides an attractive reductionist approach to investigate the molecular underpinnings of sarcomeric proteins *in isolation* – stripped of their confinement within the sarcomere cytoskeleton. The comparison between demembraned – loaded – intact membraned – (un)loaded – cardiomyocyte systems allows investigation of viscoelastic properties, in respect to the presence (or absence) of the cellular membrane, intracellular organelles and changes in ion fluxes. Explanted whole-heart approaches are the most powerful in combination with *ex vivo* hemodynamic evaluations of pressure and volume relations, which are essential to characterize the pump function of the heart in the absence of neurohormonal influences.

The extent to which the heart can relax and fill – i.e, diastolic function – is largely dependent on the compliance of the ventricular wall, in response to the magnitude and the intrinsic speed of the stretch imposed on its cardiomyocytes and the extracellular matrix (ECM). Most studies, however, mainly addressed the effects of the large sarcomeric protein titin as the central determinant of myocardial compliance and diastolic (dys)function (van Heerebeek et al., 2012; Hamdani and Paulus, 2013). Caporizzo and Prosser discuss how several non-sarcomeric components, including the microtubules and desmin, affect myocardial viscoelastic properties and the important issue of how many of the prior approaches have been either inadequate or incomplete to reveal their involvement in diastolic function. The authors provide substantial evidence to support the role of non-sarcomeric proteins as crucial components of myocardial compliance and strain, and the mechanisms through which pathophysiological alterations facilitate the transition from adaptive to maladaptive cardiac remodeling.

The ECM is an important component of the non-sarcomeric cytoskeleton that contributes to diastolic stiffness during the diastolic phase by limiting overstretch of cardiomyocytes, slippage, and tissue deformation during filling. Wittig and Szulcek review proportional changes in the major ECM components of the heart and elastic vessels. Despite identifying a surprising lack of human data, small donor numbers, and inconsistent or outdated methodology the authors found that pathological ECM ratio changes fall within a continuous scale distinguishing cardiovascular pathologies from the aged individual as well as differentiating vessels from the heart itself. The findings highlight the specific nature of biophysical microenvironmental changes and importance for experimental model systems.

Animal models are often used as final proof-of-concept. However, confounding variables are typically present in the models and often affect interpretation of the work. Craig Lygate provides a critical overview on important considerations regarding the use of gain- and loss-of function mutations in mouse models. Exemplified by the creatine kinase system, the author discusses the importance and interdependency of genetic background, promoter, group size, age, sex, diet, circadian rhythm, tissue-specificity, redundancy in signaling, compensatory adaptation, and how the choice of proper controls can impact the comparison of cardiac phenotypes *in vivo*. Such elemental issues lie at the heart of intense scientific debates surrounding animal-drug-responsiveness and the replicability of studies with heart failure animal models.

Finally, traditional approaches that investigate the pathogenesis of cardiovascular disease in small animal models typically do not allow for microscopic evaluation of *in situ* single cardiomyocytes and microvascular function. Waddingham et al. demonstrate that conventional limitations to image microvessels in small rodents (diameter 30–100 μm) can be overcome by synchrotron microangiography imaging allowing for direct investigations of small coronary microvascular function. Moreover, the authors discussed the complementarity of utilizing several synchrotron radiation imaging techniques in the same animal models to understand microlevel coronary-myocardial interactions. Uniquely, real time small-angle X-ray scattering recordings can facilitate the investigation of actin-myosin filament interactions within in the intact myofilament lattice, and provide novel insights into cardiac function *in vivo* at the sarcomere level, including a new understanding of myosin super-relaxation in pathophysiological states.

In conclusion, this Research Topic features a collection of articles that highlight essential experimental consideration well established in expert labs, but difficult to relate to until now for non-experts. By showcasing each of these methods, with their advantages and limitations, this Research Topic aims to provide a resource for scientists and help design studies that provide maximal insights on cardiovascular function.

## Author Contributions

All authors listed have made a substantial, direct, and intellectual contribution to the work and approved it for publication.

## Funding

RS is funded by the Deutsche Forschungsgemeinschaft (DFG) and the Open Access 300 Publication Fund of Charité – Universitätsmedizin Berlin. CJ is supported by the American Heart Association (20-CDA35310757), the National Institutes of Health (R35-GM142868-01), and startup funds from Mississippi State University. JP is funded by the Japan Society for Promotion of Science (KAKEN 19H03405) and Japan Agency for Medical Research and Development (AMED18072597). VS is funded by the Deutsche Forschungsgemeinschaft (DFG; Ma 2528/7-1 and SFB-894) and the German Ministry of Education and Science (BMBF).

## Conflict of Interest

The authors declare that the research was conducted in the absence of any commercial or financial relationships that could be construed as a potential conflict of interest.

## Publisher's Note

All claims expressed in this article are solely those of the authors and do not necessarily represent those of their affiliated organizations, or those of the publisher, the editors and the reviewers. Any product that may be evaluated in this article, or claim that may be made by its manufacturer, is not guaranteed or endorsed by the publisher.

## References

[B1] Hamdani N. Paulus W. J. (2013). Myocardial titin and collagen in cardiac diastolic dysfunction: partners in crime. Circulation. 128, 5–8. 10.1161/CIRCULATIONAHA.113.003437 23709670

[B2] Sequeira V. van der Velden J. (2015). Historical perspective on heart function: the Frank–Starling Law. Biophys. Rev. 7, 421–447. 10.1007/s12551-015-0184-4 28510104 PMC5418489

[B3] van Heerebeek L. Franssen C. P. Hamdani N. Verheugt F. W. Somsen G. A. Paulus W. J. (2012). Molecular and cellular basis for diastolic dysfunction. Curr. Heart Fail. Rep. 9, 293–302. 10.1007/s11897-012-0109-5 22926993

